# Preswitch Regimens Influence the Rate of Weight Gain After Switch to Tenofovir Disoproxil Fumarate, Lamivudine, and Dolutegravir (TLD): Study From an East African Cohort

**DOI:** 10.1093/ofid/ofad581

**Published:** 2023-12-12

**Authors:** Kassem Bourgi, Susan Ofner, Beverly Musick, Kara Wools-Kaloustian, John M Humphrey, Lameck Diero, Constantin T Yiannoutsos, Samir K Gupta

**Affiliations:** Division of Infectious Diseases, Department of Medicine, Indiana University School of Medicine, Indianapolis, Indiana, USA; Department of Biostatistics and Health Data Science, Indiana University School of Medicine, Indianapolis, Indiana, USA; Department of Biostatistics and Health Data Science, Indiana University School of Medicine, Indianapolis, Indiana, USA; Division of Infectious Diseases, Department of Medicine, Indiana University School of Medicine, Indianapolis, Indiana, USA; Division of Infectious Diseases, Department of Medicine, Indiana University School of Medicine, Indianapolis, Indiana, USA; School of Medicine, Department of Medicine, College of Health Sciences, Moi University Eldoret, Eldoret, Kenya; Department of Biostatistics and Health Data Science, Indiana University R.M. Fairbanks School of Public Health, Indianapolis, Indiana, USA; Division of Infectious Diseases, Department of Medicine, Indiana University School of Medicine, Indianapolis, Indiana, USA

**Keywords:** dolutegravir, efavirenz, Sub-Saharan Africa, switching antiretroviral therapy, weight gain

## Abstract

**Background:**

Switching from non-nucleoside reverse transcriptase inhibitor (NNRTI)–based regimens to dolutegravir (DTG) has been associated with greater weight gain.

**Methods:**

We conducted our analysis using a longitudinal cohort of people with HIV (PWH) in Western Kenya. We evaluated changes in the rate of weight gain among treatment-experienced, virally suppressed PWH who switched from NNRTI to tenofovir disoproxil fumarate, lamivudine, and dolutegravir (TLD). We modeled the weights pre- and postswitch using a 2-phase model with linear trend preswitch and an inverted exponential function postswitch. We estimated an 18-month excess weight gain by comparing the projected weight with that expected using the preswitch rate.

**Results:**

A total of 18 662 individuals were included in our analysis, with 55% switching from efavirenz (EFV) and 45% from nevirapine (NVP). Of the studied individuals, 51% were female, and the median age and body mass index (BMI) were 51 years and 22 kg/m2, respectively. For the overall population, the rate of weight gain increased from 0.47 kg/year preswitch to 0.77 kg/year, with higher increases for females (0.57 kg/year to 0.96 kg/year) than males (0.34 kg/year to 0.62 kg/year). The rate of weight gain for individuals switching from EFV-based regimens significantly increased from 0.57 kg/year preswitch to 1.11 kg/year postswitch but remained stable at 0.35 kg/year preswitch vs 0.32 kg/year postswitch for individuals switching from NVP-based regimens.

**Conclusions:**

Switching from NNRTI-based regimens to TLD is associated with a modest increase in the rate of weight gain, with the preswitch NNRTI being the key determinant of the amount of weight gain experienced postswitch.

Integrase strand transfer inhibitors (INSTIs) comprise an increasingly used class of antiretrovirals due to their tolerability and high efficacy [1, 2]. The use of INSTIs has been associated with an increased risk of weight gain among treatment-naïve and -experienced patients [3–7]. The World Health Organization (WHO) recently recommended using INSTIs, specifically dolutegravir (DTG), as the base component of first-line antiretroviral therapy (ART) and a preferred drug to efavirenz (EFV) [8]. Kenya is 1 of 11 countries in Sub-Saharan Africa that has introduced DTG into their national HIV treatment guidelines, with most patients being switched to or started on tenofovir disoproxil fumarate (TDF), lamivudine, and dolutegravir (TLD) combination tablets.

In a prior study from this same cohort, we found that people with HIV (PWH) starting DTG were at significantly higher risk for weight gain than those starting EFV [9]. Numerous studies have additionally emphasized that a specific subset of individuals initiating INSTI (primarily females, Blacks, and those with lower body weight) face an elevated risk of experiencing weight gain while on INSTI treatment [3, 9]. Furthermore, within the literature, weight gain has also been seen with switching to INSTI among treatment-experienced, virally suppressed PWH. A study from the AIDS Clinical Trials Group (ACTG; A5001 and A5322) showed that the rate of weight gain increased after switching to an INSTI, particularly for women [7]. Additionally, in the African Cohort Study (AFRICOS), transitioning to a DTG-based regimen was associated with an increased rate of weight gain and higher BMI [6].

It remains unclear whether the increased weight gain seen with INSTI is an off-target drug effect or whether older agents limit natural weight gain, resulting in apparently greater weight gain when initiating or switching to INSTIs [10]. Additionally, data are limited regarding whether all patients switching to INSTIs experience weight gain or whether weight gain varies by the baseline nucleoside and non-nucleoside reverse transcriptase inhibitor regimen.

In this study, we aim to assess the impact of switching from different non-nucleoside reverse transcriptase (NNRTI) regimens to TLD on weight gain in a study population from a lower middle-income country in East Africa with a high prevalence of HIV.

## METHODS

We conducted our analysis using a longitudinal cohort of PWH in Western Kenya enrolled in the Academic Model Providing Access to Healthcare (AMPATH) care and treatment program. AMPATH is the flagship program for the East Africa International epidemiology Databases to Evaluate AIDS (EA-IeDEA) consortium [11]. Clinical and demographic data were collected at enrollment and follow-up visits using standardized instruments, either transcribed into an electronic medical record (EMR) by trained data clerks or recorded directly in the EMR. These data are pooled from multiple facilities, harmonized, and undergo quality control procedures for accuracy and completeness. Under the umbrella of EA-IeDEA, this retrospective analysis was approved by the Indiana University Institutional Review Board and the Moi University Research and Ethics Committee. These regulatory bodies did not require written informed consent to use these de-identified, routinely collected, patient-level data of public health significance.

For this analysis, we identified treatment-experienced virally suppressed participants who had been on EFV- or NVP-based regimens for at least 2 years before switching to TLD and who switched at least 12 months before database closure. Participants who switched due to known viral failure, were pregnant within 2 years of the switching date, or had no recorded height were excluded. Viral failure was defined as a viral load of >1000 copies/mL within 6 months before switch. Participants were required to have at least 1 weight in the 24 months before the switch, on the date of the switch, and postswitch to better assess the drug effect on weight gain. Preswitch BMI was calculated as weight (kg) divided by height squared (m^2^) using measurements closest to the switch. Participants with preswitch BMI <18.5 kg/m2 were considered underweight, while participants with 18.5 kg/m^2^ ≤ BMI < 25 kg/m^2^ were considered to have a normal BMI. Participants with 25 kg/m^2^ ≤ BMI < 30 kg/m^2^ were considered overweight, while those with BMI ≥30 kg/m^2^ were considered obese. Preswitch CD4 count was defined as the value closest to the switching date within a window of −180 days to +7 days. Participants were grouped based on the NNRTI drug (EFV or nevirapine [NVP]) used in the 2 years before the switch.

Demographic and clinical characteristics for each group were summarized using median with interquartile range (IQR) or percentage with frequency, as appropriate. Wilcoxon rank-sum tests were used for group differences in the distributions of continuous variables; Pearson chi-square tests assessed differences in group size for categorical variables.

The primary aim of the analysis was to assess changes in the rate of weight gain for all participants after switching to TLD. We modeled weights in the 18 months preswitch, at the time of the switch, and 18 months postswitch using a 2-stage model that included a linear trend before the date of the switch and an inverted exponential function after the date of the switch. The inverted exponential function allowed for an initial increase, which leveled off over time. Predicted mean weights were calculated and plotted to show the change in mean weight pre- and postswitch.

Rates of weight gain (kg/year) were computed. To estimate 95% CIs for the rates, we generated bootstrapped samples by resampling individuals’ data with replacement 1000 times. For each bootstrap sample, we refit the model, which allowed us to generate the empirical distribution of rates. The 95% CI was obtained by selecting the 2.5th and 97.5th percentiles of the distribution. The statistical comparison of pre and post rates was assessed by determination of whether 0 was within the 95% CI of the postswitch rate minus the preswitch rate. Additionally, we estimated the excess weight gained on TLD by subtracting the expected weight gain at 18 months using the preswitch rate from the model-estimated weight at the end of the study. In the secondary analysis, we aimed to compare pre- and postswitch rates of weight gain for groups defined by sex, baseline BMI category, and by preswitch NNRTI (EFV or NVP). To account for the impact of baseline nucleoside/nucleotide reverse transcriptase inhibitors (NRTIs) on weight gain, we conducted a subgroup analysis to compare pre- and postswitch rates of weight gain for participants who maintained the same NRTI backbone (TDF/3TC) after transitioning to TLD, comparing the changes in the rate of weight gain between TDF/3TC/EFV vs TDF/3TC/NVP.

Statistical significance was determined using a *P* value <.05. The analyses were conducted using SAS/STAT software (version 9.4; SAS Institute Inc., Cary, NC, USA) and R software (R Core Team, Vienna, Austria; https://www.R-project.org/).

## RESULTS

A total of 18 662 individuals met our inclusion criteria, with 10 188 (55%) switching from an EFV-based regimen and 8474 (45%) from an NVP-based regimen. Table 1 summarizes the clinical and demographic characteristics overall and by baseline NNRTI drug. The median age was 51 years, 51% of individuals were females, and the median preswitch BMI was 22 kg/m^2^. Compared with EFV, individuals on NVP were more likely to be females (56% vs 47%) and more likely to be overweight (22% vs 18%). For the overall population, the preswitch CD4 count was 175 cells/mm3 and was significantly lower among participants switching from NVP compared with EFV (151 vs 228 cells/mm3). Participants transitioning from EFV-based regimens were primarily on TDF/3TC (85%), whereas those transitioning from NVP-based regimens were predominantly on AZT/3TC (68%).

**Table 1.** Clinical and Demographic Characteristics of all Participants Included in the Analysis and Characteristics by Switching Group

		All Subjects	EFV-Based Regimens	NVP-Based Regimens	*P* Value
Total, No. (%)		18 662	10 188 (55)	8474 (45)	<.0001
Age, median (IQR), y		51 (44–57)	50.1 (43–56)	52 (46–58)	<.0001
Sex at birth, No. (%)	Female	9591 (51)	4809 (47)	4782 (56)	<.0001
	Male	9071 (49)	5379 (53)	3692 (44)	
Preswitch BMI, median (IQR), kg/m^2^		22 (20–25)	22 (20–25)	23 (20–26)	<.0001
Preswitch BMI Category, No. (%)	Underweight (BMI <18.5 kg/m^2^)	2269 (12)	1384 (14)	885 (10)	<.0001
	Normal (18.5 kg/m^2^ ≤ BMI < 25 kg/m^2^)	11 158 (60)	6174 (61)	4984 (59)	
	Overweight (25 kg/m^2^ ≤ BMI < 30 kg/m^2^)	3768 (20)	1875 (18)	1893 (22)	
	Obese (BMI ≥30 kg/m^2^)	1467 (8)	755 (7)	712 (8)	
Preswitch CD4 count, median (IQR),^a^ kg/m^2^		175 (93–273)	228 (104–329)	151 (85–210)	<.0001
Preswitch NRTI backbone	TDF/3TC	11 216 (60)	8699 (85)	2517 (30)	<.0001
	AZT/3TC	6993 (38)	1261 (12)	5732 (68)	
	Other	453 (2)	228 (2)	225 (3)	

Abbreviations: 3TC, lamivudine; ART, antiretroviral therapy; AZT, zidovudine; BMI, body mass index; EFV, efavirenz; IQR, interquartile range; NVP, nevirapine; TDF, tenofovir disoproxil fumarate.

^a^Preswitch CD4 count data were available for 13 854 individuals (6585 in the EFV group and 7269 in the NVP group).

**Figure 1.**
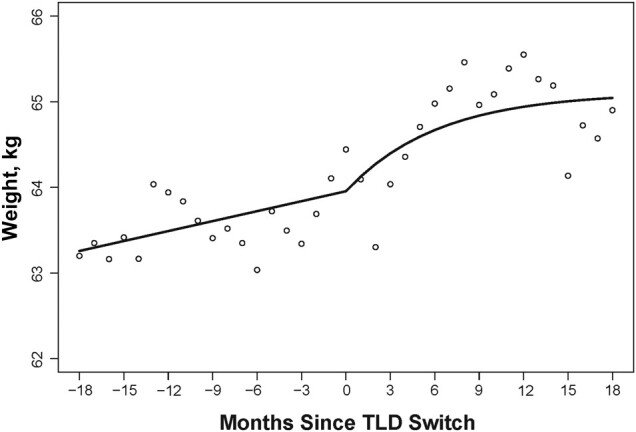
Changes in weight after switching from baseline regimen to TLD. Abbreviation: TLD, tenofovir disoproxil fumarate, lamivudine, and dolutegravir.

**Figure 2.**
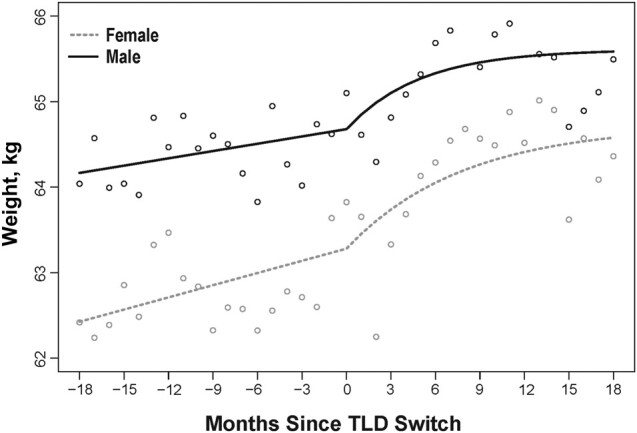
Changes in weight after switching from baseline regimen to tenofovir, lamivudine, and dolutegravir, categorized by sex. Abbreviation: TLD, tenofovir disoproxil fumarate, lamivudine, and dolutegravir.

The changes in rate of weight gain along with the 95% CI for the preswitch, postswitch, and post/preswitch rate differences, along with the estimated 18-month excess weight gain, are summarized in Table 2. Figure 1 shows changes in the rate of weight gain for the overall population, and Figure 2 highlights the changes in the rate of weight gain by sex.

**Table 2.** Rate of Weight Gain With 95% CI for Preswitch, Postswitch, and Post/Pre Rate Difference, Along With Estimated 18-Month Excess Weight Gain

		Preswitch Rate (95% CI), kg/y	Postswitch Rate (95% CI), kg/y	Post/Pre Rate Difference (95% CI)	Estimated 18-Month Excess Weight Gain, kg
Overall population		0.47 (0.39 to 0.54)	0.77 (0.66 to 0.89)	0.31^a^ (0.15 to 0.46)	0.46
Sex	Females	0.57 (0.45 to 0.68)	0.96 (0.79 to 1.22)	0.41^a^ (0.16 to 0.70)	0.59
	Males	0.34 (0.24 to 0.44)	0.62 (0.49 to 0.75)	0.28^a^ (0.10 to 0.47)	0.46
Baseline BMI Category	Underweight	−0.66 (−0.80 to −0.53)	0.76 (0.61 to 0.89)	1.42^a^ (1.19 to 1.66)	2.13
	Normal	0.37 (0.31 to 0.44)	0.58 (0.50 to 0.67)	0.21^a^ (0.09 to 0.33)	0.31
	Overweight/obese	1.12 (0.98 to 1.26)	0.27 (0.11 to 0.43)	−0.85^a^ (−1.10 to −0.59)	1.28
Switching from efavirenz	Overall	0.57 (0.46 to 0.67)	1.11 (0.97 to 1.28)	0.55^a^ (0.36 to 0.77)	0.81
	TDF/3TC/EFV	0.56 (0.45 to 0.67)	1.15 (1.01 to 1.32)	0.60^a^ (0.39 to 0.83)	0.88
Switching from nevirapine	Overall	0.35 (0.25 to 0.46)	0.32 (0.19 to 0.46)	−0.03 (−0.24 to 0.16)	−0.05
	TDF/3TC/NVP	0.25 (0.07 to 0.45)	0.67 (0.46 to 0.88)	0.42^a^ (0.11 to 0.74)	0.63

Abbreviations: 3TC, lamivudine; AZT, zidovudine; EFV, efavirenz; NVP, nevirapine; TDF, tenofovir disoproxil fumarate.

^a^Statistically significant result.

For the overall population, the model of weight gain over time shows that the overall mean weight at the switch date was 63.95 kg and participants gained 0.47 kg/year before switching to TLD. Weight gain after the switch was rapid in the first 3–4 months and then slowed so that the mean weight at 18 months was 65.11 kg. Postswitch participants gained 0.77 kg/year, which was significantly higher compared with preswitch. This difference in post/preswitch rates was 0.31 kg/year (Figure 1). The estimated excess weight gain for the overall population was 0.46 kg over the 18-month follow-up period.

The rate of weight gain after TLD switch was higher for females than males (Figure 2). The model of weight over time for females and males shows that the mean weight for females at the switch date was 63.28 kg and the mean weight for males was 64.68 kg. After a period of rapid weight gain in the few months following the switch, the model estimated that females gained 1.44 kg over the 18 months and males gained 0.51 kg less than females, but this difference was not statistically significant. The rate of weight gain for females increased 0.41 kg/year, from 0.57 kg/year to 0.96 kg/year, while the increase for males was less pronounced at 0.28 kg/year, from 0.34 kg/year to 0.62 kg/year. The estimated excess weight gain during the study period was 0.59 kg for females compared with 0.46 kg for males.

**Figure 3.**
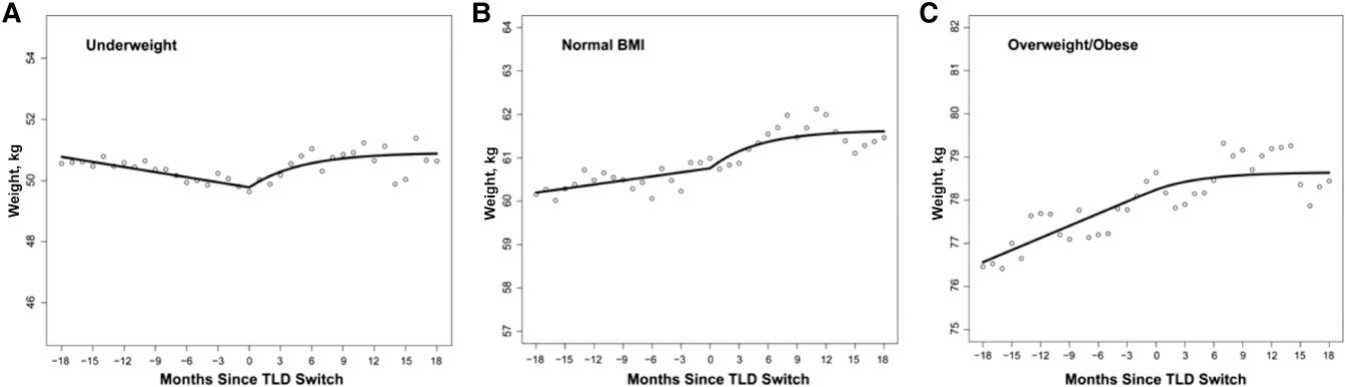
Changes in weight after switching from baseline regimen to tenofovir, lamivudine, and dolutegravir, categorized by baseline BMI category. *A*, Underweight, BMI <18.5 kg/m^2^. *B*, Normal BMI, BMI 18.5–24.9 kg/m^2^. *C*, Overweight/obese, BMI >25 kg/m^2^. Abbreviations: BMI, body mass index; TLD, tenofovir disoproxil fumarate, lamivudine, and dolutegravir.

Figure 3 shows changes in weight gain by preswitch BMI category. Compared with participants with normal and overweight/obese BMI, those who were underweight at baseline experienced the most increase in the rate of weight gain following TLD switch. The estimated excess weight gain during the study period was 2.13 kg for underweight participants, 0.31 kg for participants with normal BMI, and 1.28 kg for overweight/obese participants.

**Figure 4.**
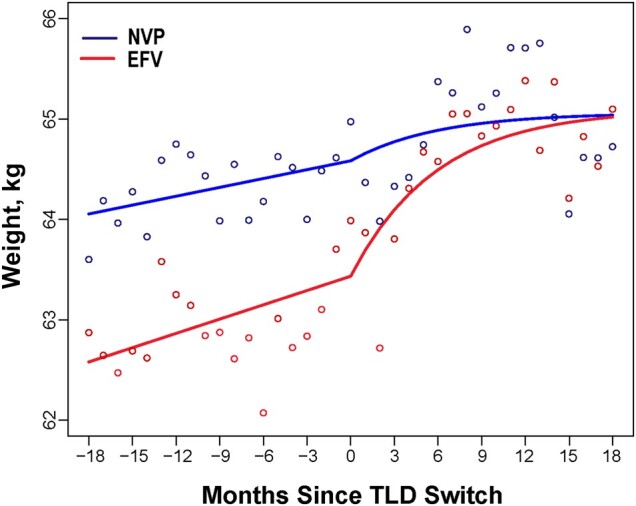
Changes in weight after switching from baseline efavirenz- or nevirapine-based regimen to tenofovir disoproxil fumarate, lamivudine, and dolutegravir, categorized by baseline non-nucleoside reverse transcriptase inhibitor drug. Abbreviations: EFV, efavirenz; NVP, nevirapine; TLD, tenofovir disoproxil fumarate, lamivudine, and dolutegravir.

Figure 4 shows changes in weight gain by preswitch NNRTI regimens. The model of weight over time by NNRTI group shows that the mean weight at the switch date was 64.58 kg for NVP and 63.43 kg for EFV. Following the switch to TLD, the mean weight for NVP increased slightly, with the mean weight at 18 months being only 0.48 kg larger than at the switch date. Weight gain for the EFV group increased rapidly in the early months, and the mean weight gain at 18 months was estimated to be 1.67 kg larger than at the switch date. Participants on EFV were estimated to have experienced on average an excessive weight gain of 0.81 kg after switching, while participants previously on NVP did not experience any excessive weight gain. The rate of weight gain for participants switching from an EFV-based regimen almost doubled, increasing from 0.57 kg/year preswitch to 1.11 kg/year postswitch. In contrast, there was no significant change in the rate of weight gain for participants switching from an NVP-based regimen, as the preswitch rate of 0.35 kg/year was comparable to the postswitch rate of 0.32 kg/year.

**Figure 5.**
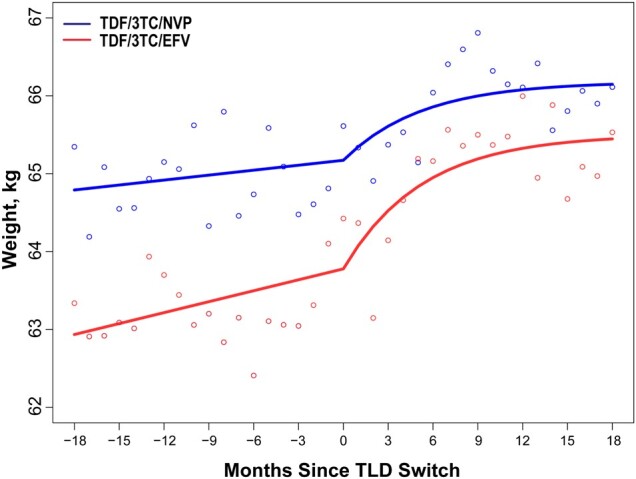
Changes in weight after switching from baseline TDF, 3TC regimen to TLD, categorized by baseline non-nucleoside reverse transcriptase inhibitor drug (EFV vs NVP). Abbreviations: 3TC, lamivudine; EFV, efavirenz; NVP, nevirapine; TDF, tenofovir disoproxil fumarate; TLD, tenofovir disoproxil fumarate, lamivudine, and dolutegravir.

Results from the subgroup analysis comparing changes in weight gain for participants who switched from TDF/3TC are shown in Figure 5. The mean weight gain at 18 months for participants who switched from TDF/3TC/EFV was estimated to be 1.7 kg compared with 1.0 kg for those who switched from TDF/3TC/NVP. Both groups experienced excess weight gain following TLD switch. For participants switching from TDF/3TC/EFV, the rate of weight gain increased from 0.56 kg/year to 1.15 kg/year, with an estimated 18-month excess weight gain of 0.88 kg. Meanwhile, for participants who switched from TDF/3TC/NVP, the rate of weight gain increased from 0.25 kg/year to 0.67 kg/year, with an estimated 18-month excess weight gain of 0.63 kg. Our subgroup analysis showed that the increase in rate of weight gain was higher for participants switching while on TDF/3TC compared with the overall population for both EFV and NVP. However, this was more pronounced among participants switching from NVP, as the group that was on TDF/3TC at baseline had an estimated 18-month excess weight gain of 0.63 kg compared with −0.05 kg for the overall NVP group.

## DISCUSSION

Our analysis based on a large cohort from Kenya demonstrated that virally suppressed individuals switching from NNRTI-based regimens to TLD experience a modest increase in the rate of weight gain postswitch. However, sex and preswitch NRTI and NNRTI drugs appear to be the key determinants of the degree of weight gain experienced postswitch. Our analysis, like several before it, highlighted higher weight gain with INSTI among women. However, we present an interesting finding of differential weight gain postswitch among individuals switching from EFV compared with those switching from NVP. Our study underscores the role of preswitch NNRTI drugs in determining the degree of weight gain postswitch, as individuals switching from NVP experienced no change in their weight gain following TLD switch, while those switching from EFV had a 2-fold increase in the rate of weight gain.

The higher rates of weight gain experienced by women compared with men following switching from NNRTI-based regimens to TLD are consistent with other studies [3, 5, 7, 9]. In the ADVANCE study of treatment-naïve participants starting DTG vs EFV in South Africa, it was noted that the DTG-associated weight gain was significantly higher in women than men [5]. It continues to be poorly understood why women are at higher risk for increased weight gain when initiating or switching to an INSTI. One recent study suggested that the DTG inhibits estrogen signaling in adipocytes, disrupting thermogenesis, leading to sex-dependent weight gain [12].

Our results suggest more significant weight gain among participants switching from EFV compared with NVP, adding to the evidence that EFV may have an inhibitory effect on weight gain. In a study using 2 cohorts of patients switching from INSTIs to EFV-based regimens, CYP2B6 genotype was associated with weight gain [13]. CYP2B6 is responsible for EFV metabolism, and genetic variations in the CYP2B6 gene can affect how quickly or slowly EFV is metabolized [14]. CYP2B6 slow metabolizers, and therefore participants with higher EFV levels preswitch, experienced higher weight gain after switching from EFV/TDF to INSTI-based regimens [13]. As noted above, in the ADVANCE study, participants starting DTG gained significantly more weight than those starting EFV [5]. However, in that study, there was a strong association between CYP2B6 genotype and weight gain. Treatment-naïve, EFV-extensive metabolizers gained a similar amount of weight compared with DTG, suggesting that the inhibitory effect of EFV on weight gain (especially among slow and intermediate metabolizers) could explain the difference in weight gain seen between DTG and EFV [15]. Additionally, in a pooled analysis of 12 prospective trials of virally suppressed participants randomized to switch or remain on a stable baseline regimen, the highest risk of weight gain was noted among participants switching from EFV [16]. All these studies highlight a possible inhibitory effect of EFV on weight gain.

Additionally, participants switching from TDF/3TC had more increases in the rate of weight gain following switch compared with the overall population. These findings highlight the weight-suppressive effect of TDF, which has been noted in several studies. In an analysis of the iPrEx trial of HIV-negative participants starting TDF for HIV prevention, participants on TDF experienced lower weight gain than those on placebo [17]. Likewise, in a systemic analysis of 7 trials using TDF/emtricitabine (FTC) or TDF in HIV-negative participants for prevention, compared with controls, participants taking TDF were more likely to experience weight loss [18]. Similar findings were noted in a recent study involving PWH, where participants in CHARACTERISE switching from tenofovir alafenamide (TAF)/FTC + DTG to TLD experienced a significant reduction in weight [19].

However, the outcomes of our subgroup analysis yielded some surprising findings. While patients who transitioned from NVP generally did not exhibit significant changes in their rate of weight gain after switching to TLD, a subgroup of patients who made this transition while already on TDF/FTC did experience some increase in the rate of weight gain. This observation suggests that, for this particular subgroup, DTG may be a contributing factor to the weight gain. Additionally, it appeared that this subgroup experienced greater weight gain than individuals who switched to TLD while on AZT/3TC. This result was unexpected as AZT has previously been demonstrated to be more weight-suppressive than TDF [20], and therefore, we would have anticipated that switching to TLD from AZT/3TC would lead to a more significant increase in weight gain compared with switching while on TDF/3TC. The reasons underlying these outcomes remain unclear, as they could be influenced by unaccounted-for confounding variables. Therefore, further studies are necessary to comprehensively comprehend the impact of background NRTIs and NNRTIs on the rate of weight gain in individuals switching to TLD.

There is an ongoing debate regarding whether INSTIs cause weight gain directly or whether older agents, such as EFV, AZT, and TDF, suppress weight, resulting in higher weight gain when initiating or switching to INSTIs [10]. A recent study assessing the role of DTG treatment intensification in virally suppressed PWH with neurocognitive impairment found no differences in weight gain between adding dolutegravir vs placebo to a stable baseline ART regimen [21]. Additionally, in a clinical trial switching patients from an integrase inhibitor–based regimen (bictegravir, tenofovir alafenamide, emtricitabine) to a combination of islatravir, an investigational nucleoside reverse transcriptase translocation inhibitor, and doravirine, an NNRTI, there was no significant change from baseline in body weight at week 48 between participants who switched off integrase inhibitors and those who remained on their baseline regimen [22].

Our observational study is the largest to date to evaluate the association between switching to TLD and weight gain in Africa. Our study includes >18 000 treatment-experienced participants switching to DTG from EFV- or NVP-based regimens. However, caution is warranted in interpreting our study findings. It is imperative to recognize that compared with randomized clinical trials, observational studies have inherent limitations in drawing generalizable conclusions, and causal inferences cannot necessarily be assured. The results might not be generalizable to all PWH, particularly those outside the East Africa region. Moreover, the majority of the individuals included in this study were female and possessed a normal BMI, potentially limiting the generalizability of these findings. In our present analysis, due to the restricted follow-up duration of 18 months, longer studies will be required to ascertain the long-term impact of switching to TLD on weight. Additionally, we did not account for concomitant medication usage, which could have contributed to weight gain (ie, psychiatric or diabetic medications), and we did not have information regarding individual-level access to food in a country with a high prevalence of food insecurity. We also did not have information regarding CYP2B6 polymorphisms; therefore, we cannot assess the preswitch EFV levels and their association with weight gain. Finally, our study did not assess clinical outcomes associated with weight gain. Future studies will be needed to assess the impact of weight gain with TLD on cardiovascular and metabolic disease incidence and progression.

Furthermore, additional studies are needed to understand whether DTG and INSTIs, in general, truly cause weight gain or whether the increased weight gain seen with these drugs is simply due to their better tolerability (fewer gastrointestinal side effects). Studies are also needed to better evaluate the mechanism of decreased weight gain seen with EFV and TDF and whether these drugs have direct anorectic effects.

In summary, our multisite cohort study demonstrates that individuals switching from NNRTI-based regimens to TLD experience modest increases in the rate of weight gain, with higher increases noted in women. However, preswitch ART appears to be a key determinant of the amount of excessive weight gain experienced after the switch. Participants switching from EFV-based regimens experience a significant increase in the rate of weight gain, unlike participants switching from NVP-based regimens, who have no change in the rate of weight gain postswitch.
